# Clinical characteristics of middle-aged and older patients with MS treated with interferon beta-1b: *post-hoc* analysis of a 2-year, prospective, international, observational study

**DOI:** 10.1186/s12883-021-02347-w

**Published:** 2021-08-23

**Authors:** Francesco Patti, Javier Nicolas Penaherrera, Lorissa Zieger, Eva-Maria Wicklein

**Affiliations:** 1 Department of Medical and Surgical and Advanced Technologies, GF Ingrassia, Neuroscience Section, University of Catania, Catania, Italy; 2 Bayer AG, Berlin, Germany; 3 Parexel Inc (at the time of this analysis), Berlin, Germany; 4 Current address: Freelance Biostatistician, Berlin, Germany

**Keywords:** Multiple sclerosis, Disease-modifying therapies, Age, Interferon beta-1b

## Abstract

**Background:**

Despite trends towards the increased age of patients living with multiple sclerosis (MS), little is known about the response of older adults with MS to disease-modifying therapies (DMTs). Thus, a *post-hoc* analysis was undertaken using data from a 2-year, international, non-interventional, prospective cohort study (NCT00787657; BEACON: BEtaferon prospective study on Adherence, COping and Nurse support) of patients above the age of 40 years with MS and starting interferon beta-1b (IFNB-1b) treatment within 6 months before study entry.

**Methods:**

Middle-aged and older patients with MS were divided into two sub-groups: 41–50 years and > 50 years. Treatment with IFNB-1b started within 6 months before study entry. Patients were followed-up for a 2-year observation period. Assessments included disease history and course, annualised relapse rate (ARR), Expanded Disability Scale Score (EDSS), treatment adherence, Hospital Anxiety and Depression Scale (HADS), and adverse events (AE).

**Results:**

At baseline, the intention-to-treat (ITT) population (*n* = 481) aged 41–50 years (*n* = 327) and > 50 years (*n* = 154), had mean (standard deviation [SD]) ages of 45.1 (2.8) and 56.2 (4.2) years, maximum age of 72 years, and duration of MS since onset of symptoms of 3.9 (5.2) and 5.9 (7.1) years, respectively. At baseline, the proportion of patients with relapsing–remitting MS (RRMS) was 96.3 and 94.9 %, and secondary progressive MS (SPMS) was 3.7 and 5.1 %, in the 41–50 and > 50 years sub-groups, respectively. The ARR in the 2 years before study start was 0.93 (0.48) and 0.86 (0.54) for the 41–50 and > 50 years groups, respectively, and decreased since study start to 0.20 (1.09) and 0.07 (0.37), respectively. The percentage of patients with anxiety and depression, as measured by HADS, were stable over the study period. Polypharmacy (five or more medications) was seen in 32.3 and 41.2 % of patients aged 41–50 and > 50 years. No unexpected AEs were reported.

**Conclusions:**

This study provides observational data on patients between 40 and 72 years of age, suggesting that IFNB-1b can be an effective and well-tolerated treatment option in MS patients of advanced age.

**Trial registration:**

ClinicalTrials.gov, NCT00787657.

## Introduction

Multiple sclerosis (MS) is a chronic immune-mediated inflammatory disorder that affects the central nervous system (CNS). In about 85 % of patients, it starts as a ‘relapsing–remitting’ MS (RRMS), typified by periodic clinical relapses usually followed by intervals of functional recovery, whilst 15 % of patients present without relapses but with a slowly progressive disease pattern called primary progressive multiple sclerosis (PPMS) [1, 2]. The physiopathology of MS is characterised by inflammatory demyelinating lesions within the CNS, owing to the infiltration of peripheral immune cells, and with axonal injury that is becoming more widespread over time [2, 3]. Most patients are diagnosed with MS in early adulthood, typically in their 20 or 30s, with a life expectancy reported to be about 7–14 years less than in the general population [4–7]. Thus, many patients with MS can typically live for many decades with this disease [4].

Since the mid-1990s, immunomodulatory disease-modifying therapies (DMTs) have been used in patients with MS, reducing the number and severity of relapses, slowing disease progression, and decreasing the number of new lesions appearing on magnetic resonance imaging (MRI) [1, 3]. As DMTs can change the pathological immune responses underlying MS, they have become standard care for the management of relapsing forms of MS [4, 8]. However, over the years since the first DMTs were introduced, there has been a demographic shift in the MS patient population, with an increasingly larger proportion of older adults living with MS than ever before. For example, in British Columbia, Canada, the peak prevalence age range for patients diagnosed with MS increased from 45–49 years in the early 1990s to 55–59 years in 2008 [9]. Likewise, data from a health administrative database from Ontario, Canada (1996–2013) showed that from 2005 onward, the age group with the peak prevalence of MS shifted from those aged 35–49 years to those aged 50 years or older [10]. Other large databases have reported similar trends [11–13]. Significant increases have occurred in the average age of patients with MS during the last two decades [11]. Primarily, this seems to be because of increased MS patient survival rates, as prevalence increased with somewhat less marked changes in MS incidence [10, 12, 14]. Aging population demographics worldwide and the widespread use of DMTs may also play a role in this phenomenon [11–13, 15]. Age is known to influence the course of MS [11], as for example, the transition to progressive disease seems to be age-related, and on average takes place during a patient’s fifth decade [16–18]. Nevertheless, a substantial proportion remain in RRMS (e.g. Tutuncu et al. reported that 38 % with RRMS did not develop progressive disease by 75 years of age) [18].

Despite the increased age of patients living with MS, very little is known about the clinical characteristics of older adults with MS or their response to DMTs [19]. In part, this is because patients above the age of 50 or 55 years with MS were excluded from pivotal clinical trials of DMTs [20–22]. Older patients are also generally under-represented in pharmaceutical clinical trials, in part owing to a higher likelihood of comorbid conditions [23]. To the best of our knowledge, minimal data from clinical or cohort studies has been published regarding the use of DMTs in older or elderly patients with MS. As such, there is a need to better understand understudied older populations with MS and their response to DMTs. Thus, we have performed a *post-hoc* analysis of data from a large international, non-interventional, prospective cohort study (NCT00787657; BEACON: BEtaferon prospective study on Adherence, COping and Nurse support), including patients with MS starting treatment with interferon beta (IFNB)-1b within 6 months before study entry. This re-analysis examined middle-aged and older patients divided into two sub-groups (41–50 years and > 50 years), with regard to their baseline characteristics as well as their clinical, neuropsychological, and safety outcomes. The sub-analysis allows us to focus in a real-world data scenario on this less-studied patient population with MS.

## Methods

BEACON (NCT00787657) was a 2-year, prospective, international, observational study designed to investigate whether nurse support and disease-related factors affect long-term adherence to IFNB-1b treatment in patients with recently diagnosed MS or clinically isolated syndrome (CIS) [24]. This study was approved by Azienda Ospedaliero Universitaria Policlinico Vittori Emanuele Catania Comitato Etica, Via Santa, and sites were requested to obtain written informed consent from each patient prior to inclusion into the study. Data were collected between June 2008 and October 2014. Patients eligible for inclusion were treated with IFNB-1b in accordance with the summary of product characteristics who had started this treatment within 6 months before study entry. Exclusion criteria were any contra-indications listed in the IFNB-1b summary of product characteristics. Study visits occurred at baseline and at 6-month intervals thereafter. The primary outcome was the proportion of patients adhering to treatment at 6, 12, 18 and 24 months. Other assessments performed at each 6-month study visit included disease history and course, annualised relapse rate (ARR), Expanded Disability Scale Score (EDSS), Hospital Anxiety and Depression Scale (HADS), MRI findings (if available), and documentation of adverse events. A *post-hoc* analysis of the trial data was performed to compare two MS patient age ranges: 41–50 years and > 50 years. By assessing MS patient characteristics and treatment response in these age ranges it was hoped to better define MS disease course and treatment outcomes in middle aged and older patients at baseline and over the 2-year observational period. The intention-to-treat (ITT) population consisted of patients with any post-baseline visit. The safety analysis population (SAF) consisted of patients with any post-baseline information or who had reported an adverse event (AE) or serious AE (SAE) already at baseline. All methods were performed in accordance with the relevant guidelines and regulations. No formal statistical comparisons were made, and thus only descriptive statistics were used (e.g. mean, standard deviation [SD]).

## Results

A total of 1545 patients (ITT population) were enrolled in 272 centres in 28 countries. The full study results have been reported previously in abstract form [24]. The *post-hoc* analysis, reported here, analysed sub-sets of the full trial dataset consisting of patients aged 41–50 years and > 50 years. The number of *post-hoc* analysis patients enrolled in the study totalled 508 patients (41–50 years, n = 343; >50 years, n = 165). At baseline, the ITT population consisted of 481 patients (41–50 years, n = 327; >50 years, n = 154), and the SAF population comprised 492 patients (41–50 years, n = 334; >50 years, n = 158). Baseline demographics for the ITT population are shown in Table 1. Overall, the age range of patients at baseline was 41–72 years in the ITT population. The proportion of patients with confirmed RRMS and SPMS diagnoses at baseline was similar for those aged 41–50 years (96.3 and 3.7 %, respectively) and > 50 years (94.9 and 5.1 %), respectively. In 113 patients, MS symptom onset occurred when over 50 years of age.

**Table 1:** Baseline characteristics (intention-to-treat population; *n*=481)

**Characteristic**	**Aged 41–50 years (*n*=327)**	**Aged >50 years (*n*=154)**
Females, %	72.7%	66.9%
Ethnicity:		
Caucasian	74.6%	73.4%
Black	2.1%	0.0%
Hispanic	6.7%	4.5%
Asian	8.9%	16.2%
other	7.6%	5.8%
Age at study baseline, mean years (SD)	45.1 (2.8)	56.2 (4.2)
Age at onset of clinical symptoms, mean years (SD)	41.6 (5.9)	50.7 (7.5)
RRMS, %^a^	96.3%	94.9%
SPMS, %^b^	3.7%	5.1%
MS duration since onset of symptoms, mean years (SD)	3.9 (5.2)	5.9 (7.1)
Relapses during last 2 years,^a^ mean number (SD)	1.7 (1.0)	1.6 (1.1)
≥2 relapses during last 2 years,^a^ %	55.6%	52.7%
EDSS, mean (SD)	2.5 (1.5)	3.1 (1.9)
EDSS ≥3, %	41.6%	52.0%
Cranial MRI performed, %	96.3%	94.0%
Contrast-enhancing lesions, %	28.8%	22.7%
DMT use prior to BEACON, %	14.1%	13.6%

^a^Before study entry.

^b^The percentages for patients with RRMS and SPMS are based on a total of 411 patients with confirmed MS (273 patients in those aged 41–50years, and 138 in those aged >50 years).

*BEACON* Betaferon prospective study on adherence, coping and nurse support; *DMT* disease-modifying therapy; *EDSS* expanded disability status scale; *MRI* magnetic resonance imaging; *RRMS* relapsing–remitting multiple sclerosis; *SD* standard deviation; *SPMS* secondary progressive multiple sclerosis

**Fig. 1:**
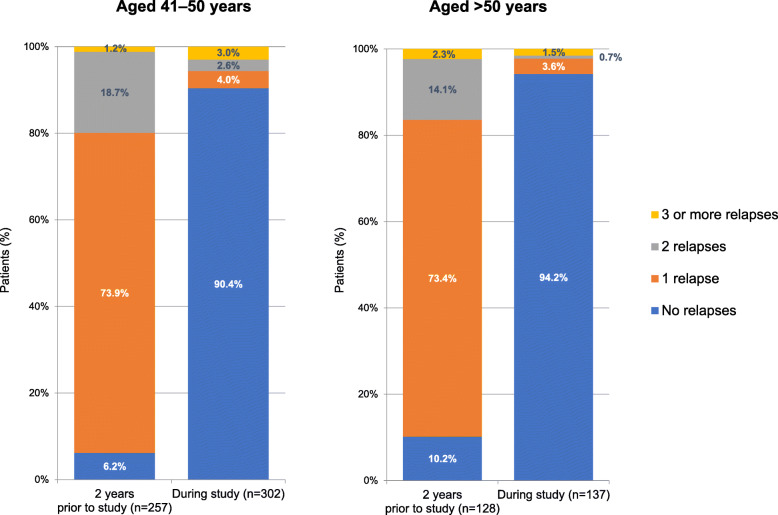
Relapses in the 2 years before the study and during the study (intention-to-treat population)

**Fig. 2:**
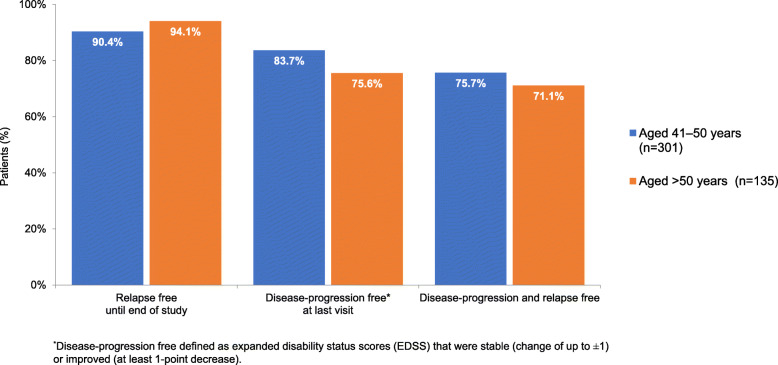
Disease progression and relapse free at last visit (intention-to-treat population), for whom these data were available (aged 41–50 years, *n* = 301; aged > 50 years, *n* = 135)

The mean ARR (SD) in the 2 years before the study (for patients with RRMS) was 0.93 (0.48) and 0.86 (0.54) for the 41–50 and > 50 years groups, respectively. During the study period, ARR (SD) corresponding values decreased to 0.20 (1.09) and 0.07 (0.37), respectively. The actual number of relapses in the 2 years before the study (for patients with RRMS) compared with number of relapses during the study are shown in Fig. 1. During the study, the proportion of patients with no relapses was 90.4 and 94.2 % for the 41–50 and > 50 years groups, respectively, compared with 6.2 and 10.2 % with no relapses in the 2 years before the study (for patients with RRMS). Disease progression and relapses for the ITT population (Table 2; Fig. 2) show that disease progression was similar and stable in the two age groups over the course of the study.

**Table 2:** Disease progression and relapses for the intention-to-treat population for whom relapse/EDSS data were available (*n*=436)

**Parameter**	**Number (%) patients**	
	**Aged 41–50 years (*n*=301)**	**Aged >50 years (*n*=135)**
EDSS score:		
improvement (≤–1)	33 (11.0%)	12 (8.9%)
stable (no change)	219 (72.8%)	90 (66.7%)
progression (≥+1)	49 (16.3%)	33 (24.4%)
Relapse free throughout the study	272 (90.4%)	127 (94.1%)
Disease-progression free^a^	252 (83.7%)	102 (75.6%)
Relapse free and disease-progression free	228 (75.7%)	96 (71.1%)

^a^Disease-progression free defined as EDSS that were stable (change of up to ±1) or improved (at least 1-point decrease) at last patient visit.

*EDSS* expanded disability status scores.

**Fig. 3:**
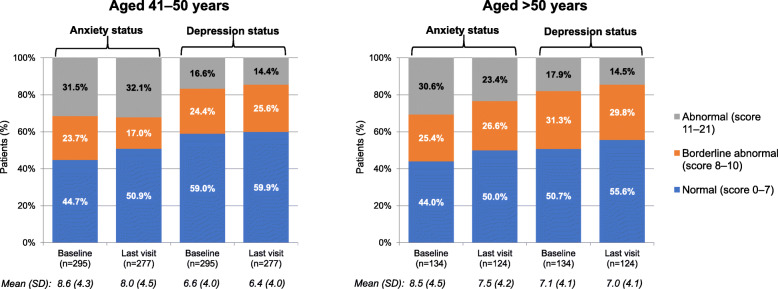
Hospital Anxiety and Depression Scale (HADS) status and mean scores at baseline and at last visit (intention-to-treat population)

Neuropsychological assessments of anxiety and depression, as determined using HADS anxiety and depression scores, showed a similar degree of stability in both age groups over the 2-year study. At last visit, mean (SD) HADS anxiety scores were 8.0 (4.5) and 7.5 (4.2) in the 41–50 and > 50 years age groups, respectively. Likewise, HADS depression scores were 6.4 (4.0) and 7.0 (4.1) in the 41–50 and > 50 years age groups, respectively. Figure 3 shows mean HADS anxiety and depression scores, as well as the proportion of patients in each age group with abnormal, borderline abnormal, or normal scores, for the ITT population.

Within the SAF population, 50.0 % (246 of 492 patients) were taking one or more additional medications (i.e. drugs other than IFNB-1b). This corresponds to 48.2 % (161 of 334) of those aged 41–50 years and 53.8 % (85 of 158) aged > 50 years who were taking additional medications. Table 3 displays the age-group related data for those taking additional medications, showing the number of additional concomitant drugs taken. The most widely used definition of polypharmacy (taking five or more drugs) [25] was applied, revealing that five or more medications (corresponding to ≥4 medications in addition to IFNB-1b) were being taken by 32.3 and 41.2 % of patients aged 41–50 years and > 50 years, respectively. Table 4 displays numbers for comorbidities relevant for patients with MS, and derived from patients comedications and their indications: cardiovascular risk factors and disorders, infections, malignancies, and autoimmune diseases other than MS. Furthermore, 45 of the patients between 41 and 50 years of age and 25 of those above 50 years were treated for depressive or anxiety disorders.

**Table 3:** Polypharmacy among patients receiving at least one medication other than interferon beta1-b (*n* = 246)

Number of additional medications^a^	Number (%) patients^b^	
	**Aged 41–50 years(*n* = 161)**	**Aged > 50 years(*n* = 85)**
1	46 (28.6 %)	12 (14.1 %)
2–3	63 (39.1 %)	38 (44.7 %)
≥4	52 (32.3 %)	35 (41.2 %)

^a^Additional medications defined as drugs other than interferon beta-1b.

^b^48.2% (161 of 334) aged 41–50 years and 53.8% (85 of 158) aged >50 years were taking additional medications within the safety population (*n*=492).

**Table 4:** Incidence of comorbidities in the total safety population (*n* = 492) and among patients taking at least one medication other than interferon beta1-b (*n* = 246)

Indication(s)^a^	Aged 41–50 years		Aged > 50 years	
	n (%) safety population (*n* = 334)	n (%) comorbid population(*n* = 161)	n (%) safety population(*n* = 158)	n (%) comorbid population(*n* = 85)
Arterial hypertension	23 (6.9 %)	23 (14.3 %)	19 (12.0 %)	19 (22.4 %)
Diabetes mellitus	5 (1.5 %)	5 (3.1 %)	3 (1.9 %)	3 (3.5 %)
Dyslipidaemia	7 (2.1 %)	7 (4.3 %)	12 (7.6 %)	12 (14.1 %)
Cardiovascular disorders, other^b^	10 (3.0 %)	10 (6.2 %)	8 (5.1 %)	8 (9.4 %)
Infection^c^	6 (1.8 %)	6 (3.7 %)	2 (1.3 %)	2 (2.4 %)
Malignancies	0 (0.0 %)	0 (0.0 %)	2 (1.3 %)	2 (2.4 %)
Autoimmune disease	13 (3.9 %)	13 (8.1 %)	5 (3.2 %)	5 (5.9 %)

^a^Additional medications taken by patients were recorded, and the comorbidity data presented here is derived from the indications for these co-medications.

^b^The category of ‘other cardiovascular disorders’ is derived from additional medications with the following indications: anti-aggregation, stroke prevention, thromboembolism, arrhythmia and vascular disease.

^c^Infection for which patients received antibiotics excluding sexual transmitted disease.

Table 5 shows safety data for the SAF population (*n* = 492) according to patients’ age group (41–50 years, *n* = 334; >50 years, *n* = 158). The number of patients (%) with any AEs was 50 (15.0 %) and 31 (19.6 %) in the 41–50 and > 50 years age groups, respectively. The number of patients (%) with the most common adverse events in the 41–50 years age group were 6 (1.8 %) with influenza-like illness, 5 (1.5 %) with headache, and 5 (1.5 %) with depression. In the > 50 years age group the most common adverse events were 5 (3.2 %) with injection-site reaction, 3 (1.9 %) with headache, and 3 (1.9 %) with depression. The number of patients (%) with any SAEs was 14 (4.2 %) and 2 (1.3 %) in the in the 41–50 and > 50 years age groups, respectively. None of the SAEs in the > 50 years age group were judged as being likely to be associated with taking the study medication, but in the 41–50 years age group, 4 patients had SAEs attributed to the study medication: 1 (0.3 %) each of congestive cardiomyopathy, acute pancreatitis, hepatotoxicity and depression. There were 5 deaths during the study period, 3 (0.9 %) in the 41–50 years age group and 2 (1.3 %) in the > 50 years age group. However, none of these deaths were judged as being likely to be associated with taking the study medication.

**Table 5:** Adverse events in the safety population (*n* = 492)

	Number (%) of patients with AEs	
	**Aged 41–50 years (*n* = 334)**	**Aged > 50 years (*n* = 158)**
Any AE	50 (15 %)	31 (19.6 %)
Any MS-related AE	22 (6.6 %)	4 (2.5 %)
Any severe AE	11 (3.3 %)	6 (3.8 %)
Any serious AE	14 (4.2 %)	2 (1.3 %)
Any AE related to study drug	32 (9.6 %)	21 (13.3 %)
Any serious AE related to study drug	4 (1.2 %)	0 (0.0 %)
Deaths	3 (0.9 %)	2 (1.3 %)
Deaths related to study drug	0 (0.0 %)	0 (0.0 %)

*AE* adverse event

## Discussion

The objective of the current study was to assess baseline characteristics, treatment responses and safety for patients with MS aged above 40, sub-grouped as either 41–50 or > 50 years, to better define the MS disease course and treatment outcomes in patients from middle age onwards. The proportion of patients with RRMS or SPMS diagnoses at baseline was 96.3 and 3.7 % for those aged 41–50 years and 94.9 and 5.1 % for those aged > 50 years, respectively. Moreover, the mean age at onset of symptoms was 50.7 years in the > 50 years age group, suggesting the presence of late-onset MS in this group. Both the 41–50 and > 50 years age groups had active disease: ≥2 relapses during last 2 years: 55.6 and 52.7 %, respectively; EDSS ≥ 3, 41.6 and 52.0 %, respectively; percentage of contrast-enhancing lesions, 28.8 and 22.7 %, respectively. On average, during a patient’s fifth decade they transition from RRMS to progressive disease [26], and this period is also associated with other changes such as an accelerated accumulation of physical disability, and a shift from active inflammatory lesions to ‘smoldering’ (chronically active or slowly expanding) plaques in the brain [13]. The fact that older patients with MS tend to have an inflammatory milieu different from their younger counterparts has given rise to a logical view that older patients might not respond to immunomodulatory agents in the same manner as younger patients [13]. Many factors other than inflammation may also influence outcomes for older patients with MS, including increased number of comorbid conditions and polypharmacy in older patients, as well as age-related immunological changes [11, 27, 28]. Furthermore, when deciding to start or continue to treat patients with DMTs, the risks as well as the benefits of any therapeutic intervention must be considered, and these choices have become increasingly complex given the lack of information and complex pathophysiological changes associated with older patients with MS. However, in the present study, it was notable that the proportion of patients taking multiple additional medications (≥4 medications in addition to IFNB-1b) was similar in the two age groups studied (32.3 and 41.2 % of patients aged 41–50 years and > 50 years, respectively), and not dissimilar to other studies. For example, Frahm et al. found that 30.3 % of outpatients with RRMS were taking at least 5 medications [28]. Moreover, a recent systematic review found polypharmacy rates between 15 and 59 % for patients with MS, and that polypharmacy correlated with comorbidities, increased risk of hospitalisation, and also increased disability, cognitive deficits, higher relapse rate and lower quality of life [29]. Cardiovascular comorbidities and diabetes have been found to increase MS disability [27]. Higher prevalence of comorbidities in MS patients leading to worse neurological, social and economic outcomes as well as to more complex treatment decisions have been published [30]. Compared with these figures, the frequency of cardiovascular risk factors and other prominent comorbidities was lower in the current study (see Table 4). One limitation of this study is that these data on comorbidities are derived indirectly from comedication records, and thus should be interpreted with caution. Undertreatment and therefore missed comorbidities may be possible in some patients.

The proportion of patients of Asian ethnicity was higher among the > 50 years population (16.2 %) compared with the 41–50 years age group (8.9 %). We can only speculate about potential reasons for this. Firstly, we would like to clarify that this was a global study that included sites in the Asia Pacific region such as China, Taiwan and South Korea. Higher percentages of Asians in the older age group may suggest a different prescription behaviour in patients above 50 years in Asia, or may be also related to a greater life expectancy in some countries included (e.g. in South Korea) than in the other regions. Finally, as the > 50 years age group is fairly small (n = 154), this makes the likelihood of an ethnically representative sample less likely than for the larger 41–50 years age group (n = 327).

Little is known about the response of older adults with MS to DMTs, despite trends towards the increased age of patients living with MS [9, 10, 12, 14], though the clinical phenotype and course of MS are known to be age dependent [16]. Whilst there have been a few studies that have described the characteristics and/or prognoses for older patients with MS, typically concerned with late-onset MS (LOMS), there is a lack of studies concerning the response to DMTs in this age group [19]. One observational study has been conducted in a small group of older patients with LOMS and RRMS (aged ≥ 50 years) treated with a DMT (n = 90), but this did not show any significant reductions in disability compared with historical controls [19]. Thus, data on the safety and efficacy of DMTs in older patients with MS are missing, indicating the need for further studies in this age group [13].

Given the current lack of data concerning the effectiveness of DMT use in older patients, decisions on whether to continue or discontinue DMT regimens in this group have become difficult, particularly in those with a stable disease course, and to date no randomised controlled trials have addressed this question. However, a prospective observational study evaluated patients who were clinically stable and taking a DMT for at least 5 years, of whom 485 stopped and 854 continued treatment [31]. Time to first relapse among between groups was similar (hazard ratio [HR] 1.07, 95 % confidence interval [CI] 0.84–1.37; *p* = 0.584), whilst there was a longer time to disability progression in patients who continued treatment than in those who discontinued (HR 1.47, 95 % CI 1.18–1.84; *p* = 0.001). This study informed subsequent guidelines from the European Committee of Treatment and Research in Multiple Sclerosis (ECTRIMS) and the European Academy of Neurology (EAN) on whether to continue or discontinue DMTs, as these state “Consider continuing a DMD if a patient is stable (clinically and on MRI) and shows no safety or tolerability issues” [32]. Nevertheless, randomised controlled trials are needed to investigate the effect of discontinuing DMTs in older stable patients, and several are ongoing but have not yet reported results, such as the DISCO-MS trial (NCT03073603) and the STOP-I-SEP study (NCT03653273).

Within the context of the data regarding ongoing treatment with DMTs in older patients, the results of the present study indicate that IFNB-1b appears to be effective and well tolerated in MS patients aged 41–50 or those older than 50 years. For example, the observed decrease in relapse rates for both age sub-groups during the study compared favourably with the number of relapses in the 2 prior years, and was also similar in the two age groups. For example, the ARR (SD) during the 2 years before study start was 0.93 (0.48) and 0.86 (0.54) for the 41–50 and > 50 years groups, respectively, and during the study period the ARR was 0.20 (1.09) and 0.07 (0.37), respectively. Reducing risk of relapse is particularly important in older patients as the ability to recover from relapse seems to decline with age [33]. Disease progression was stable in the two age groups over the course of the study. For example, the proportion of patients who were relapse free and disease-progression free throughout the study was 75.7 and 71.1 % for the 41–50 and > 50 years groups, respectively. Furthermore, neuropsychological measures of anxiety and depression showed overall stability in both age groups over the 2-year study. At last visit, mean (SD) HADS anxiety scores were 8.0 (4.5) and 7.5 (4.2) in the 41–50 and > 50 years age groups, respectively, and likewise HADS depression scores were 6.4 (4.0) and 7.0 (4.1), respectively. No unexpected adverse events were reported in this study for either age sub-group.

Given the outcomes for MS patients aged 41–50 or those older than 50 years in this real-world evidence current study, it seems that these patients benefitted from ongoing use of IFNB-1b during this 2-year observational period, with no evidence of particular increases in safety risks with greater age. These findings from a real-world setting are promising and demonstrate the positive benefit–risk ratio for IFNB-1b in this study population of middle-aged and older MS patients. However, randomised controlled trials are needed to better inform treatment decisions in this understudied age group.

## Data Availability

The raw data used for this analysis are not publicly available owing to the proprietorial nature of the data.

## Abbreviations

AE: adverse event
ARR: annualised relapse rate
BEACON: BEtaferon prospective study on Adherence, COping and Nurse support
CIS: clinically isolated syndrome
CNS: central nervous system
DMT: disease-modifying therapy
EAN: European Academy of Neurology
ECTRIMS: European Committee of Treatment and Research in Multiple Sclerosis
EDSS: Expanded Disability Scale Score
HADS: Hospital Anxiety and Depression Scale
IFNB: interferon beta
ITT: intention-to-treat
HR: hazard ratio
LOMS: late-onset multiple sclerosis
MRI: magnetic resonance imaging
MS: multiple sclerosis
PPMS: primary progressive multiple sclerosis
RRMS: relapsing–remitting multiple sclerosis
SAE: serious adverse event
SAF: safety analysis
SD: standard deviation
SPMS: secondary progressive MS
